# Intensity modulated radiotherapy with concurrent chemotherapy for larynx preservation of advanced resectable hypopharyngeal cancer

**DOI:** 10.1186/1748-717X-5-37

**Published:** 2010-05-15

**Authors:** Wen-Yen Huang, Yee-Min Jen, Chang-Ming Chen, Yu-Fu Su, Chun-Shu Lin, Yaoh-Shiang Lin, Ying-Nan Chang, Hsing-Lung Chao, Kuen-Tze Lin, Li-Ping Chang

**Affiliations:** 1 Department of Radiation Oncology, Tri-Service General Hospital, National Defense Medical Center, Taipei, Taiwan; 2 Department of Otolaryngology-Head & Neck Surgery, Tri-Service General Hospital, National Defense Medical Center, Taipei, Taiwan

## Abstract

**Background:**

To analyze the rate of larynx preservation in patients of locally advanced hypopharyngeal cancer treated with intensity modulated radiotherapy (IMRT) plus concurrent chemotherapy, and compare the results with patients treated with primary surgery.

**Methods:**

Between January 2003 and November 2007, 14 patients were treated with primary surgery and 33 patients were treated with concurrent chemoradiotherapy (CCRT) using IMRT technique. Survival rate, larynx preservation rate were calculated with the Kaplan-Meier method. Multivariate analysis was conducted for significant prognostic factors with Cox-regression method.

**Results:**

The median follow-up was 19.4 months for all patients, and 25.8 months for those alive. The 5-year overall survival rate was 33% and 44% for primary surgery and definitive CCRT, respectively (p = 0.788). The 5-year functional larynx-preservation survival after IMRT was 40%. Acute toxicities were common, but usually tolerable. The rates of treatment-related mucositis (≥ grade 2) and pharyngitis (≥ grade 3) were higher in the CCRT group. For multivariate analysis, treatment response and cricoid cartilage invasion strongly correlated with survival.

**Conclusions:**

IMRT plus concurrent chemotherapy may preserve the larynx without compromising survival. Further studies on new effective therapeutic agents are essential.

## Background

Laryngopharyngectomy followed by radiotherapy (RT)/chemoradiotherapy (CRT) has been one of treatment modalities for patients with hypopharyngeal cancer. However, it leads to the loss of a functional larynx. Larynx preservation modality for hypopharyngeal cancer has been tested in a trial conducted by the European Organization for Research and Treatment of Cancer (EORTC) Head and Neck Cancer Cooperative group [1]. It concludes that induction chemotherapy plus definitive RT offered 35% of 5-year larynx preservation rate and does not compromise survival compared with surgery. Some retrospective studies show a 5-year overall survival varying widely from 14% to 43% after RT [2-4]. However, the actual larynx preservation rate is seldom reported. Concurrent chemoradiotherapy (CCRT) has been thought to be better than sequential treatment from previous studies. Two important meta-analyses have concluded that the survival benefit from chemotherapy in head and neck cancer is based on concurrent, rather than induction use [5,6]. Nevertheless, there has been no randomized trial testing definitive CCRT versus surgery for hypopharyngeal cancer so far.

Intensity modulated radiotherapy (IMRT), a new RT technique, has the advantages of precise delivery, target conformity and normal tissue sparing. It is able to achieve a very high rate of locoregional control with less morbidity under optimal target delineation, appropriate physical quality control and accurate patient setup [7]. Although it has provided promising results in patients with other subsites of head-and-neck cancer [7-13], publications of using IMRT on hypopharyngeal cancer are rare. In our institution, CCRT has been one of the choices for resectable advanced hypopharyngeal cancer for more than 10 years and IMRT has been introduced since 2003. In this study, we analyze the rate of larynx preservation in patients of advanced resectable hypopharyngeal cancer after IMRT plus concurrent chemotherapy and compare the result with primary surgery.

## Methods

### Patients

We retrospectively reviewed medical records from January 2003 to November 2007 and identified 47 patients with histologically confirmed, previously untreated, locally advanced resectable squamous cell carcinoma of hypopharynx, who underwent primary surgery or definitive IMRT with concurrent platinum-based chemotherapy. Locally advanced resectable disease was defined as AJCC 2002 clinical stage II-IVA, excluding T1N0, small T2N0, T4b, N3, and M1 disease. Patients with T1-2N0 disease were excluded because they already had conspicuous success on larynx preservation using RT alone or CCRT, and rarely needed radical surgery. Those patients who had second primary cancer were excluded, too. The median age at diagnosis was 57, ranging from 40 to 73. Pretreatment evaluation included medical history, physical examination, complete blood counts, serum biochemistries, laryngoscopy, upper GI panendoscopy, chest X-ray, head and neck MRI and/or CT. Bone scan was conducted according to the clinical symptoms. Positron Emission Tomography was not routinely used for staging purpose. The information of advantages and disadvantages of different treatments were offered to all patients. The final treatment modalities depended on the patients' decision except for 3 patients who were assigned to CCRT; 1 with poor performance status (ECOG = 2) and 2 with severe medical comorbidity who could not undergo surgery under general anesthesia. The detailed patient characteristics were listed in Table 1.

**Table 1:** Patient characteristics.

		Treatment		
		
		Surgery	CCRT	*p*-value
		No.(%)	No.(%)	
No. of patients		14(30)	33(70)	
Sex	male	13(93)	32(97)	0.523
	female	1(7)	1(3)	
Age(y)	≥ 60	7(50)	14(42)	0.633
	< 60	7(50)	19(58)	
	Median	58	57	
	Range	43-73	40-71	
Performance (ECOG)	0	10	23	0.805
	1	4	9	
	2	0	1	
Location	Pyriform sinus	11(79)	30(91)	0.085
	Pharyngeal wall	0(0)	2(6)	
	Post-cricoid	3(21)	1(3)	
Histology grade	1-2	8(57)	20(61)	0.292
	3	6(43)	9(27)	
	NA	0(0)	4(12)	
cT	1	0	1(3)	0.795
	2	3(21)	7(21)	
	3	4(29)	6(18)	
	4a	7(50)	19(58)	
cN	0	5(36)	9(27)	0.488
	1	1(7)	7(21)	
	2a-2c	8(57)	17(52)	
Clinical stage	II	2(14)	2(6)	0.652
	III	2(14)	5(15)	
	IVA	10(71)	26(79)	
cTCI	Yes	7(50)	19(58)	0.633
	No	7(50)	14(42)	
cCCI	Yes	2(14)	7(21)	0.581
	No	12(86)	26(79)	
RT dose(Gy)	Median	62	70	
	Range	60-70	70-75	
Follow-up time(m)	Median	19.8	18.8	
	Range	6.7-67.9	1.9-72.3	

Abbreviation: CCRT, concurrent chemoradiotherapy; ECOG, Eastern Cooperative Oncology Group; NA, not available; cT, clinical T stage; cN, clinical N stage; cTCI, clinical thyroid cartilage invasion; cCCI, clinical cricoid cartilage invasion

### Surgery

Fourteen patients underwent radical surgery as the primary treatment. These included 11 patients who had total laryngectomy with partial pharyngectomy and 3 patients who underwent total laryngectomy with total pharyngectomy. Ipsilateral thyroid lobectomy was conducted in 2 patients due to suspected thyroid gland involvement. All 14 patients also had neck dissection and 3 of them underwent bilateral neck dissection. The type of neck dissection was determined by the clinical nodal status individually. The general principle was ipsilateral modified radical neck dissection or supraomohyoid neck dissection for clinical N0 disease, ipsilateral modified radical neck dissection or extended neck dissection for clinically positive-node disease. There were 21 nodes dissected on average. Pathological stages were identical to clinical stages in 10 patients. Another 4 patients had higher pathological stages than clinical stages.

### Radiotherapy technique

All patients were immobilized in supine position using custom-made thermoplastic masks. CT simulation was conducted with 3-mm slice thickness (SIEMENS Simview NT simulator). All patients in CCRT group were treated with IMRT technique. Inverse treatment planning was performed using the Plato RTS computer system version 2.6.3 (Nucletron). There were usually 6 or 7 beams with a single isocenter. The gross tumor volume (GTV) was defined as grossly visible primary tumor and metastatic lymphadenopathy on image or physical examination. The high-risk clinical target volume (CTV2) encompassed the GTV, the pyriform sinus, post-cricoid area, retropharyngeal, parapharyngeal region and bilateral level II-III nodal area. The ipsilateral level IB and V were included if clinical nodal disease was present. Four patients underwent tracheostomy before RT to prevent airway obstruction; their tracheostoma sites were included in the CTV2. The low-risk clinical target volume (CTV3) included bilateral level IV and supraclavicular areas. The planning target volume 1 (PTV1) was GTV plus a 0.6-cm margin. The PTV2, PTV3 was CTV2, CTV3 plus a 0.4-cm margin, respectively. The median prescribed dose to the PTV1, PTV2, PTV3 was 70, 60, 50 Gy, respectively. In the primary surgery group, 10 patients had postoperative CCRT or RT alone. For postoperative IMRT, the median prescribed dose to the high-risk and low-risk area was 62 and 50 Gy, respectively. The daily fraction dose to the PTV1 was 1.8-2.0 Gy, five fractions a week. All the PTVs were treated at the same time using simultaneous integrated boost (SIB) technique. The mean dose to the parotid glands was 26 Gy or lower if possible to reduce damage to salivary functions. The maximal dose of the spinal cord was kept below 45 Gy. A pair of orthogonal radiographs or images taken from Elekta electronic portal imaging device were obtained to confirm positioning accuracy before the first day of treatment. Radiotherapy was delivered with 6 MV photons from a linear accelerator (Precise, Elekta).

### Chemotherapy

Chemotherapy included cisplatin +/- 5-fluorouracil. In the CCRT arm, 15 patients had cisplatin weekly (30 mg/m^2^) for up to 8 cycles or tri-weekly (60-80 mg/m^2^) for 3 cycles. Eighteen patients had cisplatin (60-80 mg/m^2 ^day 1) + 5-fluorouracil (800-1000 mg/m^2 ^day 1 to 4) every 3 to 4 weeks. The first cycle of chemotherapy was often given in the same week as the beginning of RT. Seven patients in the primary surgery group underwent adjuvant CCRT; 4 with cisplatin alone and 3 with cisplatin + 5-fluorouracil. The protocol of chemotherapy was adjusted by the medical oncologist according to the toxicity and patients' tolerance.

### Patient follow-up and toxicity evaluation

All patients were examined weekly by laryngoscopy and physical examination during RT. Treatment response and toxicity were recorded by the radiation oncologist. After treatment, they were followed by both radiation oncologists and head & neck surgeons 1-2 months for the first 6 months, and then every 3 months for 2 years, then every 4-6 months. History taking, physical examination, serum biochemistry, treatment-related toxicity evaluation, CT or MRI of head and neck and laryngoscopy were performed in the follow-up. The toxicity grading was based on Common Toxicity Criteria for Adverse Events (CTCAE) v3.0. Treatment response was assessed by the radiation oncologists and head and neck surgeons at 1 month after completion of RT according to the finding of laryngoscopy, CT or MRI, and physical examination. Biopsy or PET was conducted for the patients whose response grading was in controversy. Complete response (CR) was defined as complete disappearance of all lesions; Partial response (PR) was at least 50% decrease in dimension; Progressive disease (PD) was 25% increase; Stable disease (SD) was neither PR nor PD. Laryngectomy-free survival referred to patients who survived at the last follow-up without laryngectomy, regardless of hypopharynx-larynx function. Functional larynx-preservation survival was defined as survival with preservation of not only an intact hypopharynx-larynx, but also normal function. Larynx preservation rate was the rate of patients who never underwent laryngectomy, regardless of survival or functional preservation.

### Statistics

Overall survival, locoregional progression-free survival, larynx-preservation survival rates were calculated with the Kaplan-Meier method, and the differences between groups in survival curves were examined using the log-rank test. All of the tests were two-sided, and p < 0.05 was considered to be statistically significant. The differences of the patient characteristics between the 2 groups were examined with Chi-square test. Multivariate analysis was conducted for significant prognostic factors with Cox-regression method. Analysis of the data was performed using SPSS 12 software.

## Results

### Survival

**Figure 1:**
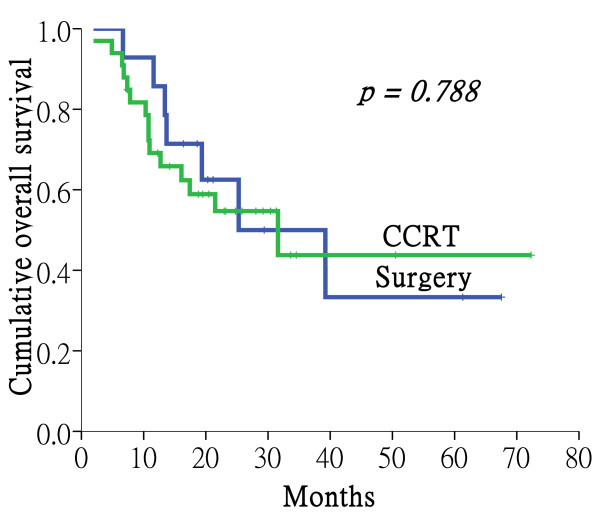
**Overall survival of the primary surgery group vs. concurrent chemoradiotherapy (CCRT) group**. The 5-year overall survival rate was 33% and 44% for primary surgery and definitive CCRT, respectively (p = 0.788).

The median follow-up was 19.4 months for all patients, 19.8 months for surgery group and 18.8 months for CCRT group, respectively. In those alive, the median follow-up time was 25.8 months, ranging from 14.2 to 72.3 months. At the time of analysis, 23 patients were alive, 2 patients were lost to follow-up, and 22 patients had died; 20 died of disease, 1 died of heart attack, 1 died of esophageal cancer. The 2-year and 5-year overall survival of all patients was 57% and 37%, respectively. The 5-year overall survival rate was 33% and 44% for primary surgery and definitive CCRT, respectively (p = 0.788, Figure 1).

The 5-year disease-specific survival rate of primary surgery and CCRT group was 33% and 56%, respectively (p = 0.970). The 5-year disease-free survival was 25% and 41%, respectively (p = 0.844). The 5-year locoregional progression-free survival was 15% and 53%, respectively (p = 0.365). Differences were not statistically significant. Loco-regional progression was the main cause of failure in both groups. The detailed patterns of failure were shown in Table. 2. Eleven patients had neck failure; 8 in the ipsilateral neck, 2 in the contralateral neck, and 1 in the tracheostoma site. All were in-field failure in the PTV2.

**Table 2:** Patterns of failure after treatment

Failure	Primary surgery No.(%)	Definitive CCRT No.(%)	Total No.(%)	*p*-value
None	3(21)	17(52)	20(43)	0.056
LR alone	6(43)	11(33)	17(36)	0.534
Distant alone	1(7)	2(6)	10(6)	0.890
LR & distant	4(29)	3(9)	7(15)	0.086
Second cancer	1(7)	5(15)	6(13)	0.452

Abbreviation: No., number; LR, loco-regional; CCRT, concurrent chemoradiotherapy

### Larynx preservation

**Figure 2:**
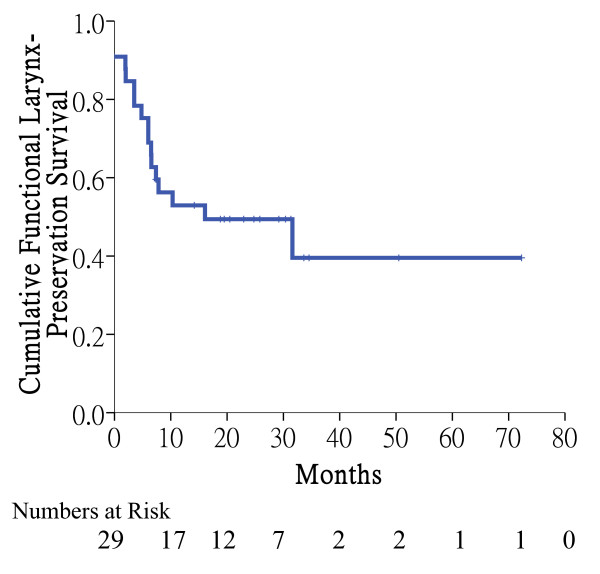
**Functional larynx-preservation survival after concurrent chemoradiotherapy**. Four patients underwent tracheostomy at initial diagnosis to prevent airway obstruction. The 5-year functional larynx-preservation survival was 40%.

After definitive CCRT, the number of patients with CR, PR, SD, and PD was 16 (48%), 15 (45%), 1 (3%), and 1 (3%), respectively. Six patients underwent salvage surgery (1 neck dissection, 5 laryngectomy with neck dissection). One of them had pathological CR. One patient who completed CCRT had local recurrence and ultimately required tracheostomy. One additional patient with fixation of his hemilarynx at diagnosis experienced bilateral vocal cord palsy 1 month after completion of RT. He subsequently needed tracheostomy. Eventually, 22 patients preserved a functional larynx. Figure 2 showed the functional larynx-preservation survival of the patients in CCRT group. The 5-year functional larynx-preservation survival, laryngectomy-free survival rate was 40%, 43%, respectively.

### Prognostic factor analysis

The result of univariate analysis of survival was shown in Table 3. Clinical T stage, thyroid cartilage invasion, cricoid cartilage invasion, worse performance status and treatment response significantly affected overall survival. For multivariate analysis, these 5 factors were included for evaluation of their effects on overall survival (Table 4.). Free of cricoid cartilage invasion and CR after treatment were significant predictors for better overall survival (p = 0.043 and < 0.001, respectively).

**Table 3:** Prognostic factors for overall survival in univariate analysis.

Factors	*p*-value	
	
	All patients	CCRT
Age ≥60 vs. <60 years	0.922	0.942
Histology Grade 1-2 vs. 3	0.995	0.768
Location Pyriform sinus vs. Pharyngeal wall vs. Post-cricoid	0.396	0.359
Stage II-III. vs. IVA	0.109	0.072
cT stage T1-3 vs. T4a	0.024	0.020
cTCI Yes vs. No	0.037	0.034
cCCI Yes vs. No	0.003	0.010
cN stage N0-2a vs. N2b-2c	0.087	0.051
Performance(ECOG) 0 vs. 1-2	0.025	0.037
Pretreatment hemoglobin ≤13 vs. >13 gm/dl	0.117	0.172
Treatment modality Surgery vs. CCRT	0.788	
Total RT day (CCRT group) <60 vs. ≥60 days		0.568
Treatment response CR vs. PR+SD+PD	< 0.001	< 0.001

Abbreviation: CCRT, concurrent chemoradiotherapy; cTCI, clinical thyroid cartilage invasion; cCCI, clinical cricoid cartilage invasion; CR, complete response; PR, partial response; SD, stable disease; PD, progressive disease

**Table 4:** Prognostic factors for overall survival in multivariate analysis.

Factors	5-year overall survival (all patients)	*p*-value	
		
		All patients	CCRT group
cT stage		0.671	0.376
T1-3	67%		
T4a	16%		
cTCI		0.815	0.621
No	68%		
Yes	16%		
cCCI		0.023	0.020
No	57%		
Yes	0%		
Performance		0.443	0.990
ECOG 0	47%		
ECOG 1-2	0%		
Treatment response		< 0.001	0.001
CR	52%		
PR+SD+PD	NA*		

Abbreviation: CCRT, concurrent chemoradiotherapy; cTCI, clinical thyroid cartilage invasion; cCCI, clinical cricoid cartilage invasion; CR, complete response; PR, partial response; SD, stable disease; PD, progressive disease; NA, not available

*The longest follow-up of this subgroup is 28 months and 2-year survival rate is 15%.

Treatment response was the most important prognostic factor. In CCRT group, the 16 patients with CR had 75% 5-year overall survival, which was significantly better than non-CR patients. All non-CR patients who did not undergo salvage laryngectomy eventually died within 2 years. Five patients who underwent salvage laryngectomy had a 2-year survival rate of 40%.

### Toxicity

Acute and late toxicities were listed in Table 5. Acute pharyngitis was the most common sequela and developed in virtually all of the patients. The rates of mucositis (≥ grade 2) and pharyngitis (≥ grade 3) were higher in the CCRT group. Since both groups used the same fractionation dose, this was probably due to the higher total radiation dose in the CCRT group (70-75 Gy vs. 60-70 Gy). Three patients in the CCRT group suffered from grade 4 leukopenia. There were 4 patients who had RT interruption more than 5 days due to toxicities (3 grade 3 leukopenia, 1 grade 3 pharyngitis). In general, CCRT was tolerable for most patients.

**Table 5:** Treatment toxicities.

	Patient number		
	
	Surgery	CCRT	*p*-value
Patients	14	33	
Acute toxicities			
Skin (≥Gr. 2)	5	6	0.194
Mucositis (≥Gr. 2)	1	13	0.027
Pharyngitis (≥Gr. 2)	9	29	0.060
Pharyngitis (≥Gr. 3)	0	10	0.020
Leukopenia (≥Gr. 3)	0	5	0.123
Anemia (≥Gr. 2)	1	11	0.060
Weight loss (≥Gr. 2)	4	13	0.480
Wound infection	3	1*	0.039
Pharyngocutaneous fistula	3	1*	0.039
Late toxicities			
Xerostomia at 1 yr after treatment (≥Gr. 2)	1	0	0.121
Neck fibrosis (≥Gr. 2)	3	3	0.246
Feeding tube-dependent	0	1*	0.510
Dysphagia (≥Gr. 2)	0	2	0.347
Carotid artery blowout	0	1*	0.510
Vocal cord palsy		4	

Abbreviation: CCRT, concurrent chemoradiotherapy

*The patients underwent not only CCRT, but also salvage surgery.

Two patients had severe late toxicities and ultimately failed to retain a functional larynx in the CCRT group. One needed tracheostomy because of bilateral vocal cord palsy. The other became feeding tube-dependent after salvage laryngectomy. Xerostomia was mild and continued to decrease over time from the end of RT. Only one patient complained of grade 2 xerostomia at 1 year after treatment. Her average dose of the bilateral parotid glands was 25.9 and 23.1 Gy.

Previous CCRT did not increase perioperative complication rate in the subsequent salvage surgery. For the six patients who underwent salvage surgery, 2 experienced surgery-related complications (1 with pharyngocutaneous fistula, 1 with wound infection). This complication rate was comparable to that of the primary surgery group. However, one patient who had T4aN1M0 disease and salvage pharyngolaryngoesophagectomy developed a carotid artery rupture 4 months after surgery. He was rescued by an emergent ligation operation. He was still alive with no evidence of disease at the last follow-up.

## Discussion

Our study shows that IMRT with concurrent chemotherapy demonstrated comparable results with primary surgery in terms of overall survival, disease-specific survival, and local control. The biggest reward is that it provides a 40% 5-year larynx preservation survival rate. Table 6 shows the retrospective treatment results including larynx preservation rate in the literature of hypopharyngeal cancer after CCRT with IMRT technique [14,15].

**Table 6:** Studies of hypopharyngeal cancer treated with IMRT.

Author (year)	Inclusion/Stage	Case no.	Follow-up (months)	Survival	Larynx Preservation
Lee et al. (2007) [14]	Retrospective review 2002-2005 Stage III-IV	11	24 (median)	53% (2-year OS)	53% (2-year LFS)
Studer et al. (2006) [15]	Retrospective review 2002-2005 T1-4N0-3	29	16 (mean)	90% (2-year DFS)	96% (LP)
this study (2010)	Retrospective review 2003-2007 T2-4aN0-2c	33	19 (median) 22 (mean)	55% (2-year OS) 44% (5-year OS) 51% (2-year DFS) 41% (5-year DFS)	54% (2-year LFS) 43% (5-year LFS) 49% (2-year FLPS) 40% (5-year FLPS)

Abbreviation: IMRT, intensity modulated radiotherapy; OS, overall survival rate; DFS, disease-free survival rate; LFS: laryngectomy-free survival rate; LP: larynx preservation rate; FLPS: functional larynx-preservation survival rate

Although CCRT is more effective for advanced head and neck cancer than RT alone, it may also be more toxic [5,16]. IMRT may spare more normal tissues, and has been shown to have decreased toxicities in head and neck cancer [17-19]. In the present study, IMRT with concurrent chemotherapy is tolerable although there are more severe mucositis and pharyngitis. The interruption of RT due to toxicities is not common. It seems that the advantage of IMRT offsets the disadvantage of CCRT.

We recommend that all potential candidates of larynx preservation should be discussed in a multidisciplinary team to assess the justification, advantages and disadvantages. Besides, optimal delineation of target volume is a requirement. Our design of the PTV described in section "Methods, Radiation technique" is relatively small. However, there has been no out-field failure in the neck. Our guideline for target contouring appears to be reasonable and may serve as a reference.

Salvage surgery is necessary for non-CR patients. No non-CR patients who did not have salvage surgery were cured in this study. Therefore, it is essential to identify the non-CR patients to CCRT as early as possible. In our practice, we determined the response after a full dose of 70 Gy. This did not interfere with wound healing after salvage surgery. A randomized study may identify those potential patients for CCRT at a lower dose as in the laryngeal cancer [20].

There are at least two limitations in this study. First, it is a retrospective study from a single institute. Non-randomization, as well as low sample size, may make selection bias and comparison statistically inherently inappropriate. Second, we add a small margin (6 mm around GTV) to create a PTV, concerning normal tissue damage. It's helpful to decrease treatment toxicities. However, this may be one of factors that compromise locoregional control.

Our IMRT using SIB technique with daily fractionation dose of 1.8-2 Gy to PTV1 results in approximate 1.5 Gy to the lower neck per day. This may be criticized for its probable radiobiological disadvantage. However, there is no in-field failure in the PTV3 in this study. In other series using IMRT with SIB in head and neck cancer, the daily dose to the lower neck is about 1.6 Gy and no higher failure rate is mentioned either [14]. There are indeed diverse dose fractionation regimens in practice of IMRT with SIB technique nowadays [21]. The long-term locoregional results in the low-risk area using different protocols are still unknown. A large prospective study with long-term follow-up is needed for creating standard regimens.

In our study, the patients have a 40% opportunity to retain their functional larynx which is an invaluable gain for every patient. This would be very cost-effective compared to the benefits of many cancer treatments that offer 10-20% locoregional control rate [22,23].

Nearly all head and neck cancer expresses EGFR and it is correlated to an unfavorable prognosis [24-26]. In a phase III trial, adding cetuximab, an EGFR inhibitor, to RT provided improvement in locoregional control and overall survival on squamous cell carcinoma of the head and neck [27]. However, only 15% were patients of hypopharyngeal cancer in that study and subgroup analysis showed no statistical benefit for them. It is being investigated in an ongoing phase III trial (RTOG-0522) comparing CCRT with CCRT plus cetuximab in patients with stage III or IV squamous cell carcinoma of the oropharynx, hypopharynx, and larynx [28].

## Conclusions

Locally advanced resectable hypopharyngeal cancer can be treated with IMRT plus concurrent chemotherapy, resulting in a 40% 5-year functional larynx-preservation survival. This combined modality, although leading to more mucositis and pharyngitis, is tolerable. However, the prognosis is still poor. Further studies on new effective therapeutic agents are essential.

## Competing interests

The authors declare that they have no competing interests.

## Authors' contributions

WYH, YMJ, CSL carried study design. WYH, CMC collected the data and performed statistical analysis. WYH, YMJ, YFS drafted the manuscript. YSL, YNC took care of the patients and helped to draft the manuscript. HLC, KTL, LPC participated in manuscript preparation and gave advice on the work. All authors have read and approved the final manuscript.
